# Takayasu’s Arteritis Presenting with Headache and Peripheral Facial Palsy: A Case Report 

**Published:** 2016-10-03

**Authors:** Maryam Sotoudeh Anvari, Farzad Masoudkabir, Kyomars Abbasi, Mohammad Ali Boroumand, Manijeh Zarghampour, Hamidreza Goodarzynejad

**Affiliations:** Tehran Heart Center, Tehran University of Medical Sciences, Tehran, Iran.

**Keywords:** *Vascular diseases*, *Hypertension*, *Headache*, *Takayasu arteritis*, *Bell palsy*

## Abstract

Takayasu’s arteritis (TA) is a rare case of granulomatous arteritis which mainly involves the aorta and its large branches. Although arterial hypertension is the most common feature of the disease in both adults and children, patients with TA may present with numerous clinical manifestations. Our patient was a 45-year-old woman, known to have hypertension from 3 years earlier following assessments made for severe headache. One year after the diagnosis of hypertension, she developed a left-sided lower motor neuron facial palsy, which was treated with oral corticosteroids (Prednisolone). Notably, the patient's headache was relieved after she took corticosteroid therapy. Transthoracic echocardiography revealed severe aortic insufficiency and aneurysmal changes in the ascending aorta, and she was referred to our center for further evaluation. In multi-slice computed-tomography angiography, significant long stenosis of the left subclavian artery was seen and the diameter of the ascending aorta was 50 mm. The patient underwent the Bentall operation. The pathologic examination of the aortic wall specimen was compatible with giant cell aortitis and more in favor of TA with the ascending aortic aneurysm. At 6months' follow-up, the patient was in good condition and had almost recovered from facial palsy.

## Introduction

Takayasu’s arteritis (TA) is a rare condition occurring mainly in women of childbearing age. It is a kind of granulomatous arteritis, which involves large- and medium-sized arteries, mainly the aorta and its large branches as well as the proximal segments of the renal, coronary, and pulmonary arteries. The walls of the affected arteries become thickened as a result of inflammatory processes. The inflammation may cause dilation of the proximal aorta. The dilation or stenosis of the involved portions of the arteries with varying degrees results in a wide variety of symptoms.^1^ Although arterial hypertension is the most common feature of the disease in both adults and children, patients with TA may present with numerous clinical manifestations.^2^^-^^4^ We describe a TA patient presenting with headache, high blood pressure, and peripheral facial palsy.

## Case Report

The patient was a 45-year-old woman, known to have hypertension of 3 years' duration following assessments made for severe headache. Based upon the medical records and history, at the time of diagnosis, the blood pressure was measured just in one limb and no evaluation was done on the other limbs. At that time she underwent transthoracic echocardiography (TTE), which revealed normal findings except for mitral valve prolapse associated with mild mitral regurgitation and moderate aortic insufficiency (AI). However, it seems that the cardiologist who conducted the TTE failed to evaluate the aortic root specifically and simply started medical management of hypertension. One year after the diagnosis of hypertension, she developed left-sided lower motor neuron facial palsy, which was treated with oral corticosteroids (Prednisolone). Notably, the patient's headache was relieved after she took corticosteroid therapy. Recently, she underwent another TTE, which demonstrated severe AI and aneurysmal changes in the ascending aorta. Her cardiologist referred the patient to our hospital for further evaluation.

The patient had no history of Kawasaki’s disease in childhood, and she reported no cardiovascular or rheumatologic diseases before the diagnosis of hypertension. Her family history was also unremarkable. However, she had a history of urticaria, mild asthma, and also skin reaction to Trimethoprim/Sulfamethoxazole. On examination at our center, a blood pressure of 160/110 mmHg in the upper limbs was recorded and the pulses were equal in the upper limbs. The femoral pulses were normal at presentation and continued to remain so. Fundoscopy revealed normal findings, and there was no arthropathy. Laboratory findings showed an elevated erythrocyte sedimentation rate of 55 mm/1h. In multi-slice computed-tomography angiography, the coronary arteries were normal, significant long proximal stenosis of the left subclavian and some irregularity in the left common carotid artery were seen, and the diameter of the ascending aorta was 50 mm, which were altogether in favor of TA. 

**Figure 1:**
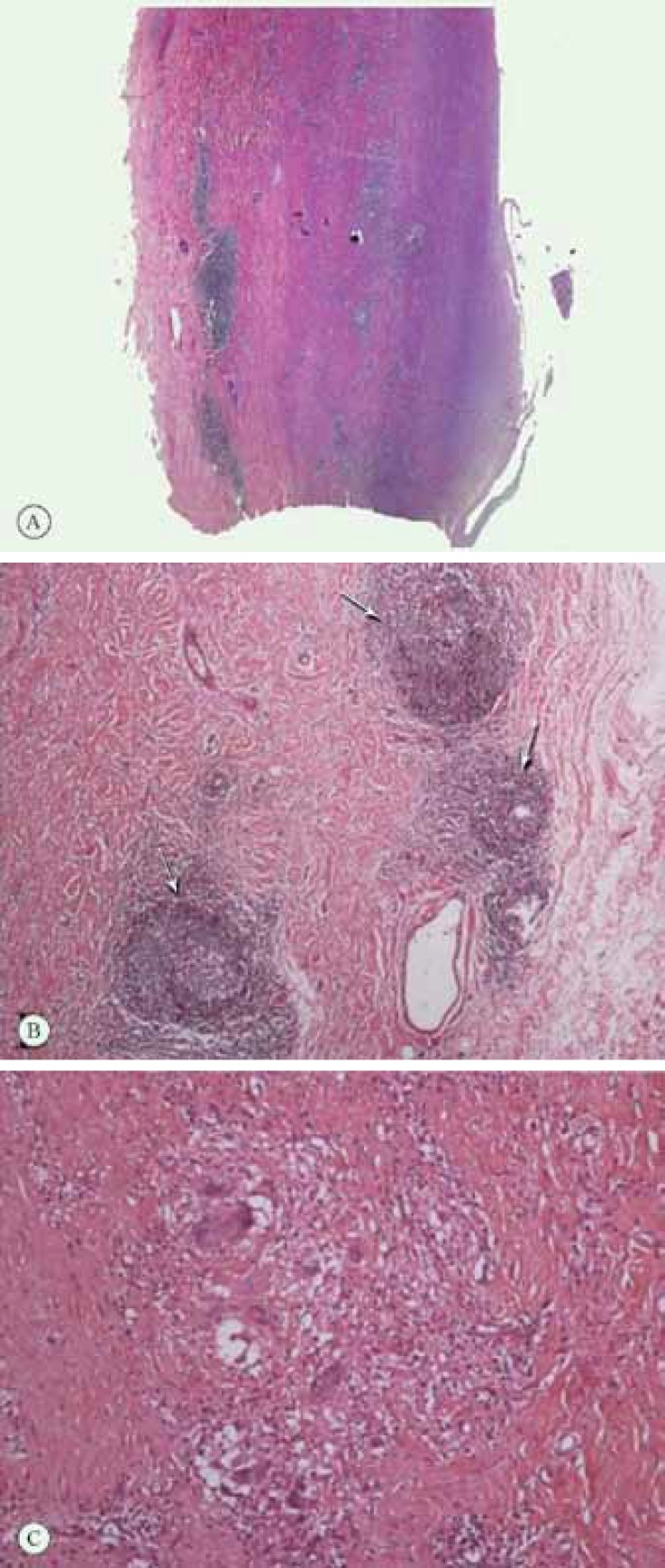
Pathologic examination of the patient’s aortic valve in hematoxylin and eosin (H & E) stain.

The patient underwent the Bentall operation, during which the aortic valve was replaced with a composite valve and hemiarch replacement was done. Specimens, including the aortic valve and aortic wall, were also sent for pathology. Pathologic examination showed aortic valve myxomatous changes compatible with AI as well as marked thickening of the intima, media, and adventitia of the ascending aortic wall and medial cystic degeneration. In addition, there was prominent lymphocyte infiltration in the adventitia and outer media as well as granulomatous inflammation with less frequent multinucleated giant cells (Figure 1). All told, the findings were compatible with giant cell aortitis and more in favor of TA with ascending aortic aneurysm. At 6 months' follow-up examination, the patient was in good condition and had almost recovered from facial palsy.

A) Full thickness cross-section of the aorta in low-power field consists of intimal fibrous hyperplasia, granulomatous reaction in the media and adventitia, and adventitial sclerosis (original magnification × 2)

B) Three granulomas at the medio-adventitial junction (original magnification × 10)

C) Multinucleated giant cells in granuloma (original magnification × 40)

## Discussion

The rarity of TA and its nonspecific clinical manifestations predispose to a late diagnosis and delayed treatment. The clinical course of TA is composed of an early active inflammatory phase and a late chronic phase.^5^^, ^^6^ The active inflammatory phase lasts for weeks to months and may have a remitting and relapsing course. It should be highlighted that the acute phase does not occur in all patients and the correct diagnosis of TA is seldom made in the early phase. The late chronic phase is caused by the arterial stenosis and/or occlusion and ischemia of organs. In our patient, the initial inflammatory phase of TA did not occur prominently and the onset of her disease was an unexplained headache which led to accidental detection of systemic hypertension. TA must be considered in all young patients with unexplained hypertension. Moreover, it should be kept in mind that blood pressure or pulse asymmetries are seen in 60 to 80% of TA patients and that their absence cannot exclude the diagnosis.^7^

Our patient also developed left-sided lower motor neuron facial palsy one year following the diagnosis of hypertension. Our extensive literature search yielded no report on the association between TA and Bell’s palsy. However, peripheral facial palsy as the initial manifestation of severe systemic hypertension has been reported in 3-17% of cases, mainly in children.^8^ Although the exact mechanisms to explain such a relationship remain to be elucidated, small hemorrhages into the facial canal (just like retinal hemorrhage) and neural partial necrosis may be possible explanations.^9^

## Conclusion

Regardless of the underlying mechanisms, clinicians should be aware of the possible association between Takayasu’s arteritis and Bell’s palsy when dealing with a young patient complaining of facial palsy.

## Notes:

***This paper should be cited as: ***Sotoudeh Anvari M, Masoudkabir F, Abbasi K, Boroumand MA, Zarghampour M, Goodarzynejad H. Takayasu’s Arteritis Presenting with Headache and Peripheral Facial Palsy: A Case Report. J Teh Univ Heart Ctr 2016;11(4):195-197.
